# Intracranial Solitary Fibrous Tumors With Glandular and Papillary Structures: A Case Report

**DOI:** 10.1002/ccr3.72746

**Published:** 2026-05-25

**Authors:** Tingting Xu, Shasha Zheng, Leiming Wang

**Affiliations:** ^1^ Department of Pathology Xuanwu Hospital Capital Medical University Beijing China; ^2^ Department of Pathology Central Hospital Affiliated to Shandong First Medical University Jinan Shandong Province China; ^3^ Department of Radiology Xuanwu Hospital Capital Medical University Beijing China

**Keywords:** NAB2, next‐generation sequencing, solitary fibrous tumor, STAT‐6

## Abstract

Solitary fibrous tumor (SFT) is a fibroblastic neoplasm with NAB2 and STAT6 gene fusion as well as STAT6 nuclear expression. Uncommon variants of intracranial SFTs, such as glandular and papillary structures, are extremely rare. We present a rare SFT case in a 64‐year‐old female demonstrating unprecedented glandular structures and extensive myxoid stromal changes. This morphologic divergence underscores the imperative for molecular validation in SFTs with atypical histologic features. Our findings advocate a refined diagnostic protocol: RNA‐based next‐generation sequencing (RNA‐NGS) must be prioritized in STAT6‐immunopositive central nervous system (CNS) mesenchymal tumors when DNA‐NGS fails to identify the pathognomonic NAB2::STAT6 fusion, as demonstrated by the resolution of diagnostic ambiguity through detection of a cryptic NAB2‐exon4::STAT6‐UTR fusion in this case.

## Key Clinical Message

Intracranial solitary fibrous tumors (SFTs) are rare. This case highlights the significance of unprecedented glandular structures in an SFT harboring a NAB2::STAT6 fusion. Such morphologic divergence underscores the imperative for molecular validation in SFTs presenting with atypical histologic features.

**Abbreviations**
H&E: Hematoxylin and eosin
MRI: Magnetic resonance imaging
NGS: next‐generation sequencing
SFT: Solitary fibrous tumor

## 1 Introduction

Intracranial Solitary fibrous tumors (SFTs) are a rare type of mesenchymal neoplasms defined by NAB2::STAT6 fusions, accounting for only < 1% of all primary central nervous system (CNS) tumors [1]. While uncommon variants (e.g., myxoid, lipomatous) are documented, glandular differentiation remains unreported [2]. Here, we report a challenging case of intracranial SFT with unique glandular/papillary structures and myxoid stroma, initially lacking STAT6 fusion on DNA‐NGS but confirmed via RNA sequencing. This case illustrates diagnostic challenges and underscores RNA‐NGS in resolving ambiguous CNS tumors.

## 2 Case History

**FIGURE 1:**
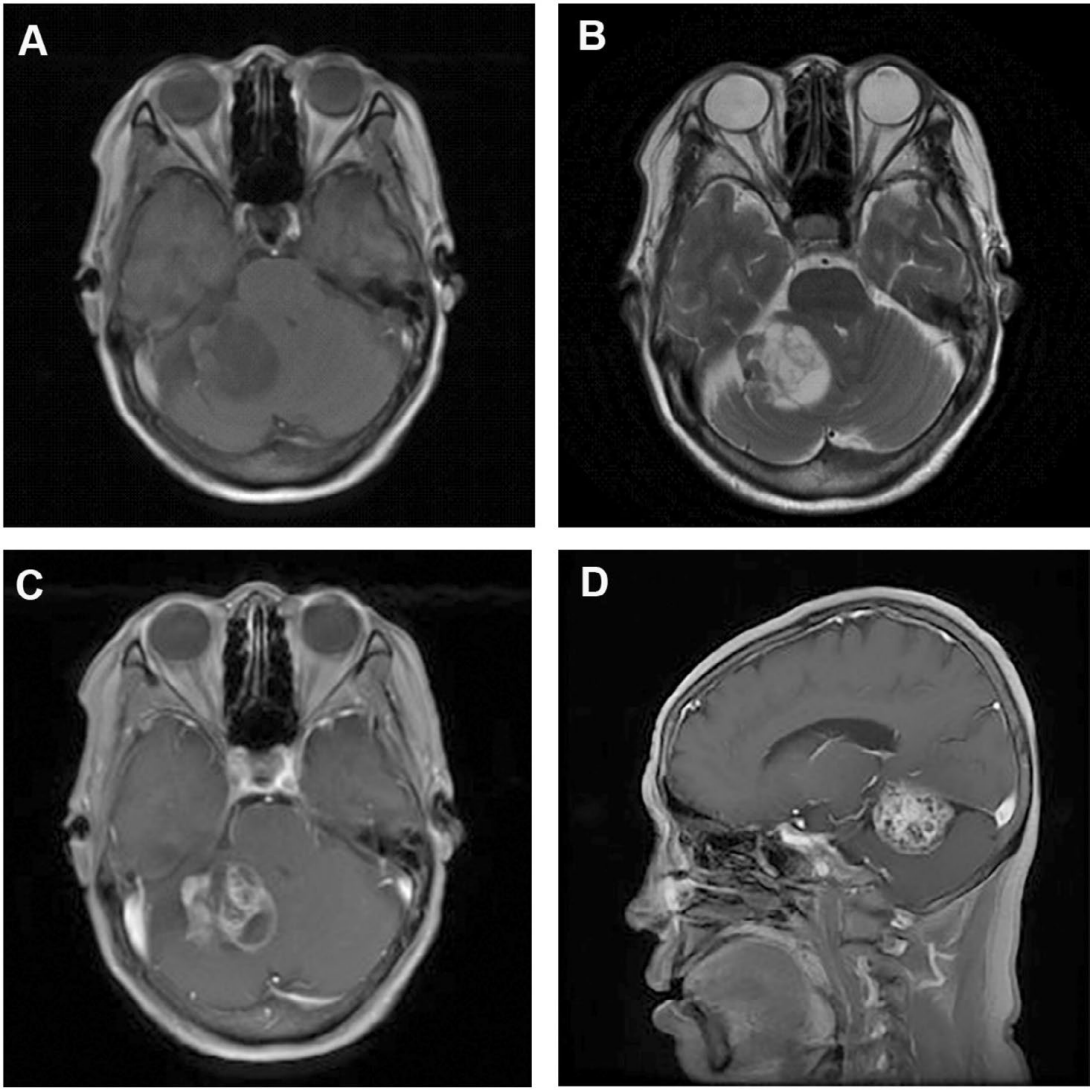
Cranial MRI showed a mass located in the tentorium cerebelli with heterogeneously isointense T1‐weighted (A) and T2‐weighted (B) image signals, and marked heterogeneous contrast enhancement on axial (C) and sagittal (D) T1‐weighted MRI with contrast enhancement.

A 64‐year‐old female patient developed gait unsteadiness and began experiencing dizziness 3 months ago. Cranial MRI shows a mass, measuring 4.5 × 4.2 × 3.8 cm, located in the right medial temporal lobe and hippocampal region. The mass showed heterogeneously isointense T2‐weighted and T1‐weighted image signals, with marked heterogeneous enhancement and thickening of the meninges adjacent to the tumor (Figure 1). Intraoperatively, the tumor was adherent to the tentorium, and the entire tumor along with the adherent portion of the tentorium was resected.

## 3 Differential Diagnosis and Investigations

**FIGURE 2:**
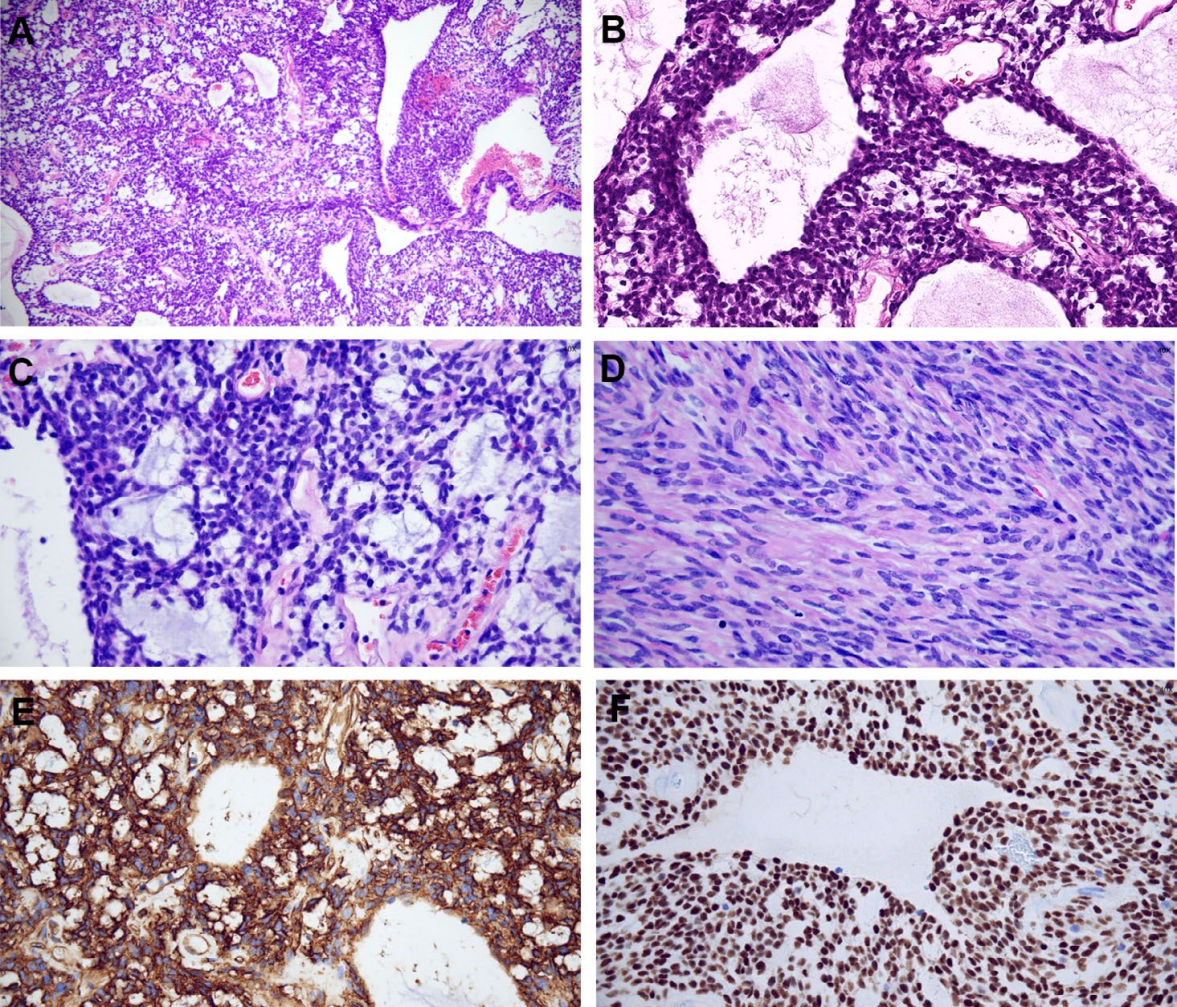
H&E staining showed glandular and microcystic structures (A, B). Myxoid stroma also can be seen (C). The tumor cells are spindle shape and arranged in storiform pattern (D). Immunohistochemistry showed the tumor cells to be strongly and diffusely positive for CD34 (E), and STAT‐6 (F).

**FIGURE 3:**
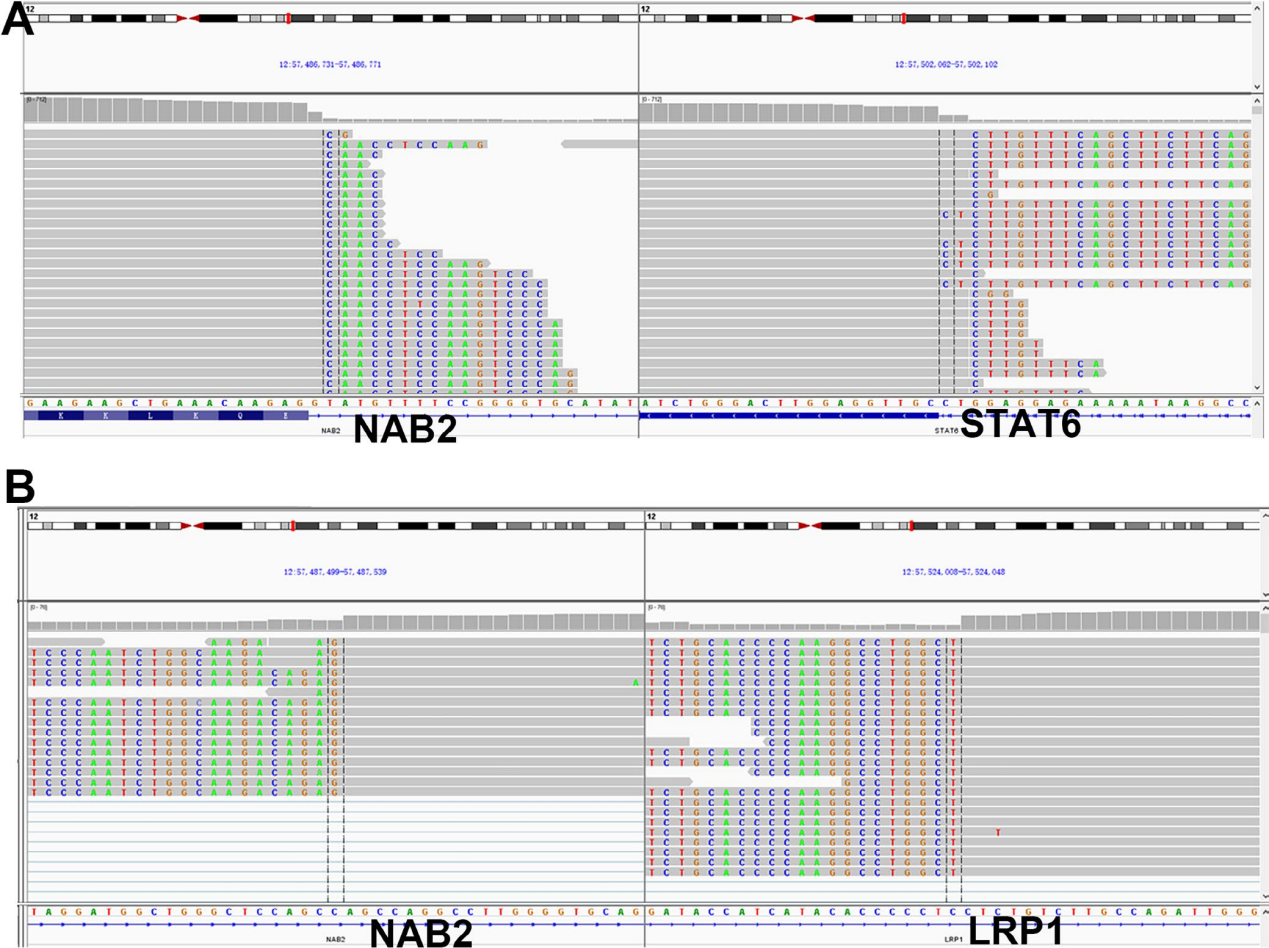
RNA‐NGS revealed NAB2::STAT6 (NAB2‐exon4::STAT6‐5′UTR) fusion (A, on the left is the NAB2 gene, and on the right is the STAT6 gene) and LRP1::NAB2 (LRP1‐exon1::NAB2‐exon7) fusion (B, on the left is the NAB2 gene, and on the right is the LRP1 gene) in the tumor.

On histopathological examination tumor cells are spindle to ovoid in shape, mainly arranged in glandular, microcystic and papillary structures (Figure 2A,B). Myxoid stroma also can be seen in some areas (Figure 2C). The surrounding tumor cells are dense and loose alternately. The tumor cells are spindle shape and arranged in storiform or whorled patterns in the dense area (Figure 2D). The stroma contains collagen fiber bundles of different thickness and staghorn vessels. Mitotic figures are commonly observed. Necrosis have not been identified. The tumor cells strongly expressed for Vimentin, CD34 (Figure 2E), STAT‐6 (Figure 2F) and BCL‐2, without immunopositivity for CK, EMA, SSTR‐2, SOX‐10, and GFAP. The Ki‐67 proliferation index is 30%. ATRX, H3K27me3, INI‐1, and BRG‐1 expression are preserved. DNA‐based next‐generation sequencing (NGS) revealed LRP1‐NAB2 (LRP1:exon1‐NAB2:exon7) fusion, TERT c.‐124C> T promoter mutation, NAB2 c.1105G> A (p.E369K) mutation, and ERCC4 c.1905‐2A> G mutation. RNA‐NGS revealed fusions between NAB2::STAT6 (NAB2‐exon4::STAT6‐5′UTR) fusion and LRP1::NAB2 (LRP1‐exon1::NAB2‐exon7) fusion (Figure 3A,B).

## 4 Conclusion and Results

According to the 2021 WHO CNS classification (Mitotic figures are 6/mm^2^ and without necrosis) [1, 3]. The final pathological diagnosis is Solitary fibrous tumor (SFT), classified as CNS WHO Grade 2. The patient did not receive adjuvant treatment after the operation because of gross total resection of the tumor and WHO Grade 2. She had a disease‐free survival time of 6 months.

## 5 Discussion

Solitary fibrous tumors (SFTs) are mesenchymal neoplasms driven by NAB2::STAT6 gene fusion [1]. While typically involving the lower limbs, head/neck, or retroperitoneum, intracranial SFTs are rare and predominantly affect individuals aged 50–70 years. These slow‐growing, often asymptomatic tumors are usually detected incidentally on imaging and favor meningioma‐associated sites (e.g., tentorium cerebelli, falx cerebri). In this case, the tumor was located in the right tentorium cerebelli region, with imaging characteristics suggestive of meningioma establishing the provisional clinical diagnosis. Classic SFT morphological features typically exhibit a “patternless” growth pattern with alternating loose and dense areas, tumor cells with mild morphology, spindle‐shaped and short spindle‐shaped, featuring thin‐walled, horn‐like blood vessels, and collagen fiber bundles of varying thickness in the stroma. Rare histological forms can occur in individual cases, such as melanocyte differentiation, papillary and lipomatous changes, and varying degrees of myxoid stroma in some cases [2]. However, as far as we know, glandular structures in SFT only have been reported in one case, which was described as gland‐like spaces [2]. The appearance of glandular and papillary structures increases the difficulty of pathological diagnosis, which requires an exception for metastatic carcinoma or mesenchymal tumors with glandular structures. Our case presented with a large number of glandular structures and myxoid stroma. Immunohistochemical staining showed that tumor cells expressed STAT‐6, Vimentin, CD34, and BCL‐2. Simultaneous increased mitotic figures are present in cell‐dense areas, but without necrosis. According to the 2021 WHO classification of central nervous system tumors, the histological grading of SFT was Grade 2 [1, 3].

The NAB2‐STAT6 fusion gene caused by 12q13 chromosomal inversions is a hallmark of solitary fibrous tumors (SFTs) [4]. Nuclear STAT6 immunoreactivity reliably indicates this fusion and serves as a specific diagnostic marker for SFT. In this case, DNA‐based NGS identified an LRP1‐exon1::NAB2‐exon7 fusion and TERT c.‐124C> T promoter mutation but failed to detect the STAT6::NAB2 fusion. However, given the distinct nuclear expression of STAT6, RNA‐based NGS was subsequently performed. This revealed both the LRP1‐exon1::NAB2‐exon7 fusion and an additional NAB2‐exon4::STAT6‐UTR fusion, which had not been previously reported. Therefore, in cases with definitive nuclear STAT6 positivity where STAT6::NAB2 fusion is not initially detected by DNA‐NGS, RNA‐based NGS sequencing is strongly recommended. Notably, this case also harbored the TERT c.‐124C> T promoter mutation. Studies suggest that hotspot mutations in the TERT promoter are closely associated with poor prognosis in SFTs [5]. Although this patient has a prolonged disease course, no tumor progression has been observed in the current six‐month follow‐up, and continued close monitoring is advised.

## 6 Conclusion

The reported SFT case exhibited histopathological features of glandular structures and myxoid stroma. Importantly, while DNA‐NGS did not detect the NAB2::STAT6 fusion, RNA‐NGS confirmed an NAB2‐exon4::STAT6‐UTR fusion. This underscores the necessity of prioritizing RNA‐NGS in diagnostically challenging mesenchymal tumors of the central nervous system (CNS), particularly those with nuclear STAT6 immunopositivity. Such approaches are critical to avoid misclassification and ensure accurate molecular characterization.

## Author Contributions

**Leiming Wang:** data curation, funding acquisition, investigation, writing – review and editing. **Tingting Xu:** data curation, methodology, writing – original draft. **Shasha Zheng:** methodology, resources, writing – original draft.

## Funding

Beijing Nova Program, Grant/Award Number: Z201100006820149.

## Ethics Statement

Written informed consent for research was obtained from the individual included in the case report.

## Consent

Written informed consent for publication and use of data were obtained from the patient.

## Conflicts of Interest

The authors declare no conflicts of interest.

## Data Availability

I confirm that my article contains a Data Availability Statement even if no data is available (list of sample statements) unless my article type does not require one (e.g., Editorials, Corrections, Book Reviews, etc.).

## References

[1] D. N. Louis , A. Perry , P. Wesseling , et al., “The 2021 WHO Classification of Tumors of the Central Nervous System: A Summary,” Neuro‐Oncology 23, no. 8 (2021): 1231–1251. 34185076 10.1093/neuonc/noab106 PMC8328013

[2] Z. Yao , H. Wu , Y. Hao , et al., “Papillary Solitary Fibrous Tumor/Hemangiopericytoma: An Uncommon Morphological Form With NAB2‐STAT6 Gene Fusion,” Journal of Neuropathology and Experimental Neurology 78, no. 8 (2019): 685–693. 31271432 10.1093/jnen/nlz053

[3] V. Matthijs , R. Beckers , C. V. Broecke , et al., “Central Nervous System Solitary Fibrous Tumors: Case Series in Accordance With the WHO 2021 Reclassification. Framework for Patient Surveillance,” Acta Neurochirurgica 166, no. 1 (2024): 414. 39417883 10.1007/s00701-024-06304-7

[4] D. R. Robinson , Y. M. Wu , S. Kalyana‐Sundaram , et al., “Identification of Recurrent NAB2‐STAT6 Gene Fusions in Solitary Fibrous Tumor by Integrative Sequencing,” Nature Genetics 45, no. 2 (2013): 180–185. 23313952 10.1038/ng.2509 PMC3654808

[5] A. Bahrami , S. Lee , I. M. Schaefer , et al., “TERT Promoter Mutations and Prognosis in Solitary Fibrous Tumor,” Modern Pathology 29, no. 12 (2016): 1511–1522. 27562490 10.1038/modpathol.2016.126 PMC5731237

